# Clinical Efficacy of Topical or Oral Soy Supplementation in Dermatology: A Systematic Review

**DOI:** 10.3390/jcm12124171

**Published:** 2023-06-20

**Authors:** Nicole Natarelli, Nimrit Gahoonia, Jessica Maloh, Raja K. Sivamani

**Affiliations:** 1 Morsani College of Medicine, University of South Florida, 560 Channelside Drive, Tampa, FL 33602, USA; natarellin@usf.edu; 2 College of Osteopathic Medicine, Touro University, 1310 Club Dr., Vallejo, CA 94592, USA; ngahooni@student.touro.edu; 3 Integrative Skin Science and Research, 4825 J St., Sacramento, CA 95819, USA; malohjessica@gmail.com; 4 Pacific Skin Institute, 1451 River Park Drive, Suite 222, Sacramento, CA 95815, USA; 5 College of Medicine, California Northstate University, 9700 W Taron Dr., Elk Grove, CA 95757, USA; 6 Department of Dermatology, University of California-Davis, 3301 C St. 1300, Sacramento, CA 95816, USA

**Keywords:** *Glycine max*, soy, isoflavones, soybean, aging, collagen, hydration, hair, acne, eczema

## Abstract

Soybean, a legume native to Southeast Asia, serves many nutritional and medical purposes due to its rich source of phytochemicals and its antioxidant activity. Many animal and in vitro studies have demonstrated its potential impact on dermatologic health. The objective of this review is to investigate the clinical response of soy-based oral supplementation or topical application on dermatologic outcomes. A systematic review of studies assessing soy supplementation or application was performed in January 2023. Databases included PubMed, Embase, Cochrane, and Natural Medicines, and studies assessing any formulation that included soybean or associated products were included. Thirty studies met the inclusion criteria and are included in the review; 13 of these studies assessed oral supplementation and 17 assessed topical application. Topical and oral supplementation demonstrated efficacious results for a variety of dermatologic parameters, including chronological or photoaging parameters, skin barrier status, hydration, hyperpigmentation, dermal network composition, erythema, hair and nail parameters, acne lesion counts, and vulvar lichen sclerosis scores. Factors associated with aging, such as wrinkle area and depth, were most frequently assessed among the studies, and both topical and oral studies demonstrated efficacy. Effects are likely mediated by dermal compositional changes, such as increased collagen and/or elastic fiber numbers. Transepidermal water loss measurements, an indicator of skin barrier status, were frequently obtained among the studies, although improvement was more likely achieved with topical application compared to oral supplementation. The results of this review highlight the utility of soy-based products for a variety of dermatologic applications, although future studies are required to determine optimal formulations and application routes for intended outcomes.

## 1. Introduction

Soybean, also known as *Glycine max*, is a species of legume native to Southeast Asia and is typically grown in tropical areas. It is a popularly produced commodity globally as it serves many nutritional and medicinal purposes. Soybean contains high contents of protein, fiber, and fat and is low in carbohydrates. Additionally, it contains a rich content of beneficial phytochemicals, such as isoflavones, and offers antioxidant activities. Due to its many attractive properties, soybean consumption has been well researched for its health impact and has demonstrated anticarcinogenic, immunomodulatory, and lipid-lowering effects [1]. The isoflavone component is uniquely similar to the structure of estrogen, and consequently has been discovered to have mild estrogen-like effects [2]. As such, it has been referred to as a phytoestrogen. Thus, many studies have highlighted its potential role in preventing postmenopausal climacteric symptoms and decreasing breast cancer risk [2]. Furthermore, a specific soy isoflavone, genistein, has been shown to have neuroprotective effects and reduce carbohydrate digestion, thereby having therapeutic potential in the management of stroke, amyotrophic lateral sclerosis, and diabetes [1].

In the realm of dermatology, genistein has been suggested to inhibit the unwanted activation of protein kinases that would otherwise lead to collagen and elastin degradation [3]. For instance, mice supplemented with soy isoflavones demonstrated lower levels of collagen-destructing metalloproteinases [4]. In addition, the phytoestrogen effects of soybeans have been suggested to improve skin aging, especially among women undergoing menopause [5]. One study conducted with human fibroblast cultures showed that the application of equol, a derivative of soybean, had comparable effects to that of 17-beta estradiol, with both agents demonstrating increased collagen and elastin, and decreased metalloproteinases in fibroblast cultures [6].

Overall, the literature demonstrates promising effects of soybean for skin health and skin appearance. Coupled with a growing interest in integrative medicine and nutrient-based interventions, a more detailed understanding of the relationship between soybean and dermatological health is warranted. In this systematic review, we summarize findings from clinical trials on the use of oral or topical soy supplementation in dermatology to provide clinically relevant information for its use.

## 2. Materials and Methods

A PubMed, Embase, and Cochrane search was performed in January 2023 with the following keywords: “Soybeans” OR “Soy Foods”, “Soy Milk”, “Soybean Oil”, “Soy Isoflavones”, “Soybean Isoflavones”, “Soy Flavonoids”, or “Soybean Flavonoids”) and “Hair”, “Skin”, “Acne Vulgaris”, or “Dermatology”. Reports with relevant titles and/or abstracts were retrieved for a full-text review. Any human in vivo study assessing the effect of topical or oral soy-based supplementation on any dermatologic outcome was included. Two authors independently conducted a full-text review, and questions regarding inclusion were brought to a third author. Additional articles were retrieved via handsearching and from the Natural Medicines Research Collaboration. Two authors collectively retrieved data from each report. Quality assessment was conducted with Jadad scoring, which scores methodological quality based on randomization, masking, and the accountability of all subjects [7]. Studies were grouped for qualitative synthesis based on the measured outcome. The heterogeneity of results and evidence quality was further discussed in the discussion section via a qualitative comparison of study factors and populations. This review was not registered and a review protocol was not prepared.

## 3. Results

### 3.1. Literature Search

**Figure 1:**
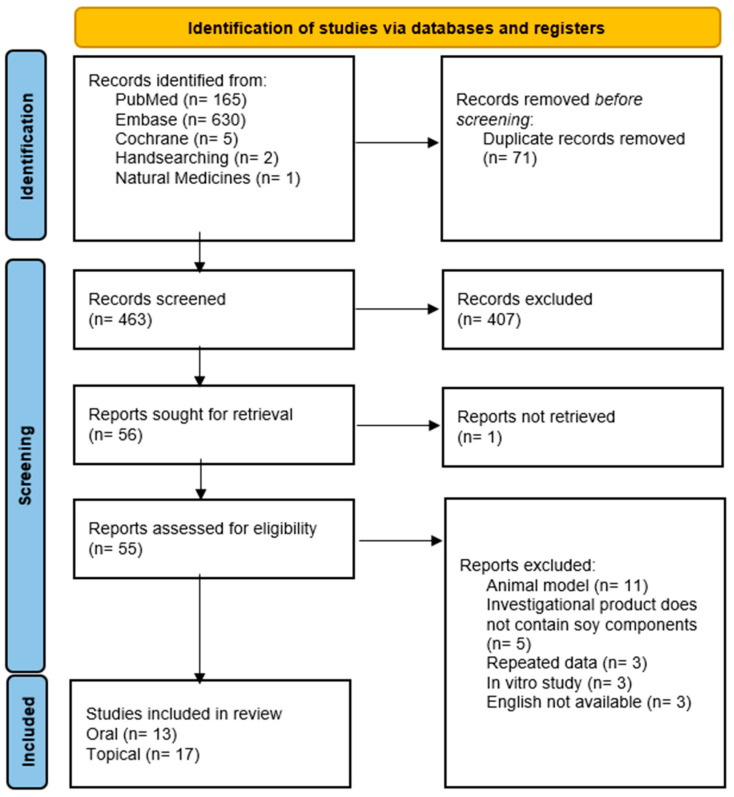
PRISMA flow-diagram of search process. The search strategy was initially conducted with three databases, although two studies were retrieved via handsearching and one study was retrieved from Natural Medicines. Following the removal of duplicate records (71), 463 records were screened. In total, 407 records were excluded based on title and abstract, and 56 full records were sought for retrieval. In total, 55 records were retrieved, of which 25 were excluded for various reasons. Briefly, 30 studies were ultimately included in the review, 13 studies assessing oral supplementation and 17 studies assessing topical application.

Our search yielded 630 articles results from Embase, 165 from PubMed, and 5 from Cochrane. Seventy-one duplicate reports were removed. Of the remaining 463 articles, 55 full texts were retrieved, of which 25 were excluded for various reasons highlighted in Figure 1. Two studies were retrieved via handsearching, and one study was retrieved from Natural Medicines Research Collaboration. Thirty studies were included in the efficacy analysis, including 13 oral studies and 17 topical studies.

### 3.2. Efficacy of Oral Soy Supplementation

In total, 13 studies assessed soy-based oral supplementation on various dermatologic parameters, including cutaneous aging, skin hydration, hair and nail appearance, and acne vulgaris lesions (Table 1). Many studies evaluated soy-based supplementation in combination with various other ingredients, such as vitamins, minerals, and fish oil, hindering the ability to assess the efficacy of soy supplementation in isolation. However, while studies assessed different products for varying periods of time, all trials observed significant improvement in at least one measured parameter when using soy supplementation compared to control supplementation and/or compared to baseline values. In total, 11 studies were randomized controlled trials (RCTs), with an average Jadad score of 3.64. The two remaining studies were open-label, and uncontrolled. Since Jadad scoring ranges from 0 to 5, studies scoring 3 or greater are considered good-quality trials. Collectively, the included studies are of adequate evidence quality.

#### 3.2.1. Cutaneous Aging and Skin Condition

Among studies assessing the effects of oral soy-based supplementation, outcomes associated with cutaneous aging were the most frequently reported. In 2006, authors assessed the effect of a soy-based dietary supplement on skin aging in 80 post-menopausal women [8]. In total, 38 women received the supplement and 42 received a placebo tablet, and each group consumed two tablets twice daily for 6 months. Treatment tablets consisted of soy extract, fish protein polysaccharides, vitamins C and E, zinc, and extracts from white tea, grape seed, tomato, and chamomile; total soy intake was equivalent to 350.0 mg per day. Clinical assessments and photographs were used to assess a variety of skin parameters. At month 6, the treatment group depicted a significantly greater improvement in forehead (9.1% reduction from baseline), periocular (14.8%), and perioral (11.8%) wrinkles; facial skin laxity (18.4%), sagging (12.5%), and mottled hyperpigmentation (15.2%); under eye dark circles (19.2%); and decolletage (8.1%) and hand crepiness (18.4%) compared to those parameters in the control group based on clinical assessment (*p* < 0.05). Photo evaluation revealed significant improvement in forehead (7.1%) and periocular (12.0%) wrinkles, facial mottled hyperpigmentation (13.3%), and overall appearance of the face (10.9%) in the treatment group compared to the control group (*p* < 0.05) [8]. Overall, dietary supplementation with the soy-based product improved the appearance and condition of the skin among post-menopausal women, although other included ingredients in the treatment group hindered the ability to draw conclusions about the impact of soy supplementation alone.

A 2007 RCT investigated the effect of soy isoflavone aglycone-containing food on skin aging parameters [9]. Thirteen women were randomized to the test food containing 40 mg soy isoflavone, while another thirteen women were randomized to receive a matched placebo. Supplementation occurred daily for 3 months. Authors observed a significant improvement in fine wrinkles (*p* < 0.05) and malar skin elasticity (*p* < 0.05) with the treatment group relative to those parameters under the placebo condition at month 2. In addition, there was significant intragroup improvement in skin microrelief at the lateral angle of the eyes with treatment (*p* < 0.05) at month 2, but no intergroup significant differences were observed for this outcome measure [9].

A distinct RCT (2007) assessed daily soy supplementation (20 g soy protein and 160 mg isoflavones) on various skin, hair, and nail parameters [3]. Forty women with mild to moderate photoaging were randomized to receive once daily supplementation for 6 months, or to continue with regular, self-selected dietary intake. Among those receiving the soy supplement, significant improvement was observed in facial skin flaking (*p* = 0.028), discoloration (*p* = 0.045), and overall appearance (*p* = 0.05) at month 3 relative to those parameters at the baseline. Following the full six months of supplementation, significant improvement was observed in facial skin wrinkling (*p* = 0.004), discoloration (*p* = 0.016), and overall appearance (*p* = 0.0001). In this same study, the investigators also assessed hair and nail outcomes; the results are outlined in Section 3.2.3.

In 2009, daily supplementation with a 100 mg isoflavone-rich concentrated soy extract for 6 months was studied for various skin parameters in an open-label, uncontrolled study [10]. Of the 29 post-menopausal women who completed the study, 23 subjects showed a 9.46% increase in epidermal thickness (*p* < 0.01). Dermal collagen increased in 25 subjects, and elastic fiber number increased in 22. However, authors noted a 41.3% increase (*p* < 0.01) in skin wrinkling after 6 months of treatment. Despite the increase in wrinkling, authors concluded that taking a concentrated soy extract for 6 months provides benefits including increased thickness of the epithelium, increased concentrations of collagen and elastic fibers, and increased number of subcutaneous vessels [10]. Authors postulate that such effects may protect the skin against UV radiation.

A 2012 study evaluated the effects of natural 5-equol supplementation, developed via the fermentation of a whole soy germ, on skin aging parameters among postmenopausal Japanese women [11]. Briefly, 34 women received daily 10 mg supplementation (EQL10), 33 received daily 30 mg supplementation (EQL30), and 34 women received placebo supplementation. Both 5-equol treatment groups depicted significant reductions in wrinkle area compared to that in the placebo group (*p* < 0.05). However, the percent change in wrinkle area increased overtime within the EQL10 group, but interestingly with the higher dosage of the EQL30 group, this intragroup increase was not observed. In addition, the EQL30 group, but not the EQL10 group, showed a significant difference in mean wrinkle depth compared to that in the placebo group (*p* < 0.05). There were no significant differences observed in other skin parameters including skin hydration, transepidermal water loss (TEWL), and elasticity [11]. These results suggest a dose-dependent efficacy response for wrinkle area and depth with 5-equol supplementation.

In 2014, authors assessed the effect of a 14-week daily oral cocktail supplementation containing soy isoflavones, lycopene, and vitamin C and E with a fish oil capsule on wrinkle reduction in post-menopausal women [12]. The 166 subjects enrolled were randomized to three groups: placebo (*n* = 55), treatment group 1 (*n* = 51) and treatment group 2 (*n* = 53). Treatment 1 contained a greater dose of isoflavone (70 mg vs. 40 mg), lycopene (8 mg vs. 3 mg), vitamin C (250 mg vs. 180 mg), and vitamin E (250 mg vs. 30 mg) compared to treatment 2. While the mean R3z value, indicating wrinkle depth, increased in the placebo group, there were no significant changes in R3z values at the final timepoint for either treatment group. The change was significantly greater in the placebo group compared to that in treatment 1 (*p* = 0.0045) and compared to that in treatment 2 (*p* = 0.0081). No significant differences between groups were observed for measures of skin firmness, elasticity, TEWL, and hydration. However, punch biopsy analysis revealed that 17.3% of individuals in treatment group 2 exhibited an increase in collagen compared to 3.6% of individuals in the placebo group (*p* = 0.0259) exhibiting this. Treatment group 1 was not assessed for changes in collagen levels [12]. These results suggest that the soy isoflavone-based oral supplement may reduce or prevent the worsening of wrinkle depth, perhaps mediated by an increase in collagen for some individuals.

A 2018 RCT evaluated the effects of an oral supplement composed of fermented soymilk (FSM) with isoflavone aglycones and a probiotic, *Lactobacillus casei Sherota (LcS)*, on skin parameters and gut microbiome composition [13]. Briefly, 60 premenopausal women were randomly assigned to drink 100 mL of the treatment supplement or unfermented soymilk control drink. The study consisted of a 4-week pre-intake period, 8-week intake period, and 4-week post-intake period. Urinary isoflavone levels, fecal microbiota, and facial skin self-questionnaires were administered and assessed throughout the study. Urinary isoflavone levels were significantly increased compared to those at the baseline in both groups during the 8-week intake period (*p* < 0.001), with no significant difference between the groups. The facial skin questionnaires assessed overall satisfaction, dryness, moisture, elasticity, pigmentation and coarseness on a 5-point Likert scale, with higher scores corresponding to a more favorable skin condition. During the intake period, the FSM group displayed significantly higher questionnaire scores of each parameter compared to the pre-intake period. However, no significant difference was observed between groups in with the results of the questionnaire. Changes in the gut microbiome were assessed and only the FSM group displayed a significantly greater abundance of *Lactobacillaceae* during the intake period relative to baseline (*p* < 0.001). Significant decreases in *Enterobacteriaceae, Porphyromonadaceae*, and *Ruminococcaceae* abundance levels were observed during the intake period compared to those at the baseline in the FSM group (*p* < 0.05) [13]. The authors concluded that probiotic supplementation with fermented soymilk can beneficially alter the gut microbiome and result in improved skin parameters. One limitation of this study is that both groups received some form of soymilk and both groups had an augmentation in their urinary isoflavone levels. This may explain why there was not a difference in the self-reported parameters between the two groups. Therefore, it is not certain that there was a true control in this study. Future studies should limit soy or soy isoflavone intake to just one of the groups.

A 2021 study assessed the effect of a soybean-containing oral supplement on dermatologic parameters and antioxidant status in post-menopausal women [14]. The supplement consisted of a blend of *Glycine max*, *Cimicifuga racemose*, *Vitex agnus-castus*, and *Oenothera biennis* extracts, while the appearance and odor-matched placebo supplements contained soybean oil. A total of 41 women in the treatment group and 50 women in the placebo group underwent once daily oral supplementation for 12 weeks. At week 12, the treatment group was found to have a significant improvement and large effects sizes in skin elasticity (Cohen’s *d* = 1.56), roughness (*d* = 1.53), smoothness (*d* = −1.33), scaliness (*d* = −0.80), and wrinkle density (*d* = −1.02) compared to the placebo group [14]. However, as the treatment supplement contained a mix of herbal components and the placebo pill contained soybean oil, it is difficult to determine the effects specifically resulting from soy supplementation in this study.

Despite the favorable effects on skin condition and cutaneous aging noted by prior studies, a 2021 study found no significant difference between treatment and control groups in skin autofluorescence, an indicator of advanced glycation end products, which are contributors of aging [15]. Authors assessed an oral supplement containing 98% S-equol (isoflavone derivative), 2% daidzein, 0.2% glycitein, and 0.1% genistein extracted from fermented soybeans. Briefly, 27 postmenopausal women were randomized to the treatment group while 30 women consumed a placebo supplement. In each group, supplements were taken once daily for 3 months. There were no significant differences found between groups for metabolic and aging biomarkers, or for skin autofluorescence. Further analysis was then performed on skin autofluorescence based on equol supplementation and innate equol production status. Here, equol producers were defined as those with a urinary equol level of 1.0 μM or greater. Skin autofluorescence improved in 3/18 participants (16.7%) with no equol exposure; 7/20 participants (35%) with extrinsic exposure; 3/8 (37.5%) participants with intrinsic exposure; and 3/4 (75%) participants with intrinsic and extrinsic exposure [15]. A greater proportion of individuals with both intrinsic and extrinsic exposure demonstrated improvement compared to those in all other groups. However, a larger trial with greater sample sizes in each group is necessary.

#### 3.2.2. Skin Hydration

A 2015 RCT assessed the effects of a cocktail containing barley and soybean compared to a placebo cocktail on skin hydration among 65 healthy adults with dry skin [16]. Thirty-two subjects were randomized to the treatment cocktail, in which they consumed 3 g daily for 2 months. Hydration under dead skin was measured via Corneometer CM825. Authors observed a significant increase in hydration on the face and the forearm with the treatment compared to that with the placebo (*p* < 0.05 at 2, 4, and 8 weeks) [16]. However, another RCT found no significant difference in skin hydration between those supplemented with 5-equol developed via fermentation of a whole soy germ compared to those supplemented with a placebo tablet [11]. Similarly, a there was also a RCT that found no difference in skin hydration between the soy isoflavone-based treatment group and the control group [12].

#### 3.2.3. Hair and Nails

A 2007 RCT assessed daily soy supplementation (20 g soy protein and 160 mg isoflavones) on various skin, hair, and nails parameters [3], previously discussed in Section 3.2.1. Among those receiving the soy supplement, authors observed a significant improvement in hair roughness (*p* = 0.004), dullness (*p* = 0.048) and overall appearance (*p* = 0.005) at six months. Significant improvement was also noted for hair roughness and overall appearance at three months (*p* = 0.041 and *p* = 0.016, respectively). While no significant nail changes were observed at three months, soy supplementation resulted in a significant improvement in nail roughness (*p* = 0.017), ridging (*p* = 0.006), flaking (*p* = 0.049), splitting (*p* = 0.007), and overall appearance (*p* = 0.008) at 6 months [3].

#### 3.2.4. Acne

A 2015 RCT investigated the effects of isoflavone supplementation on acne lesion counts among 25 female patients with acne vulgaris [17]. Subjects were randomized into 5 groups for soy isoflavone supplementation: 40 mg, 80 mg, 120 mg, 160 mg, or placebo taken daily for one month, which corresponds to the epidermal regeneration time [17]. A significant decrease in mean total acne lesion counts was observed among those supplemented with 160 mg daily (*p* < 0.05). Other treatment groups did not show a significant change from the baseline. This suggests that a dosage of 160 mg of isoflavone supplementation is needed to reduce acne vulgaris lesion counts, but it is important to note that the very small sample size within each treatment group reduces the robustness of results.

The same authors then evaluated the effects of 160 mg supplementation vs. the placebo control for 3 months among 40 female patients with acne vulgaris [18]. Acne lesion numbers significantly decreased from the baseline in the treatment group (*p* < 0.05) and non-significantly decreased in the control group (*p* > 0.05). The treatment group demonstrated a mean lesion count of 110.8 ± 47.46 and 34.0 ± 24.82 before and after 3-month treatment, respectively, indicating a mean lesion count decrease of 76.8 (69.3% decrease). In contrast, the control group depicted a mean lesion count decrease of 0.2 (0.2% decrease), with a significant difference being found between the groups (*p* = 0.001). In addition, authors measured dihydrotestosterone (DHT) levels before and after treatment, observing a mean 97.4 ± 320.72 pg/mL increase in the control group vs. a 169.5 ± 62.43 pg/mL decrease in the treatment group [18]. Authors suggest that the clinical efficacy of soy isoflavone supplementation on acne lesion counts may be mediated by antiandrogenic effects.

#### 3.2.5. Vulvar Lichen Sclerosis

A 2015 study provided supplementation with avocado and soybean extracts (ASE) in a topical and oral format to 23 subjects with mild to moderate vulvar lichen sclerosis [19]. Subjects were instructed to apply the topical ASE cream to the affected area for 6 months and take two oral 300 mg ASE for the first 3 months of the study. Global subjective scores (GSS) and global objective scores (GOS) were determined at 3 and 6 months. At the end of the study period, 100% of subjects reached GSS50 and 70.6% reached GSS75, while 88.9% reached GOS50 and 72.2% reached GOS75. In addition, a Friedman test was utilized to determine mean itching, burning, and dyspareunia values, all of which significantly decreased (*p* < 0.00001 for itching and burning; *p* = 0.003 for dyspareunia) [19]. The results indicate therapeutic effects of ASE for vulvar lichen sclerosis, although it is difficult to determine whether topical or oral supplementation is beneficial when used independently, or if there is a synergistic effect at play. To better understand the effects of the treatment interventions, a larger sample size and control groups are warranted for further analysis.

### 3.3. Efficacy of Topical Soy Application

Briefly, 17 studies assessed topical soy-based applications on various dermatologic parameters, including cutaneous aging, dermal network composition, skin barrier status, dryness, eczema, and erythema index following ultraviolet B (UVB) irradiation (Table 2). Similarly to oral supplementation, many treatment products included other ingredients, including herbal extracts and extraneous lipids, reducing the ability to assess the efficacy of soy alone. All studies, except for one [20], demonstrated improvement in at least one measured outcome. In total, 10 studies were self-controlled, and 6 studies were open-label and uncontrolled. Only one study was an RCT, with a Jadad score of 4.

#### 3.3.1. Cutaneous Aging and Hyperpigmentation

In 2007, a double-blind RCT evaluated a soy moisturizer containing soybean trypsin inhibitor (STI) and Bowman-Birk protease inhibitor (BBI), both serine protease inhibitors found in soybeans, on pigmentation and aging parameters of photoaged skin [21]. The serine protease inhibitors have been shown to inhibit melanosome phagocytosis via protease-activated receptor 2 (PAR-2), and preclinical studies have highlighted their skin-lightening potential [21]. In this study, 31 women were randomized to the active study moisturizer, and 32 women were assigned a vehicle control moisturizer. Subjects applied their respective moisturizers twice daily for 3 months. Roughness (tactile), mottled hyperpigmentation, lentigines, blotchiness, dullness (lack of brightness/clarity), and fine lines were graded on a nine-point scale graded by dermatologists and global assessments of overall skin texture, overall skin tone, and overall skin appearance were assessed on a nine-point scale as well. Following treatment, authors observed a significant improvement in mottled pigmentation, blotchiness, dullness, fine lines, overall texture, skin tone, and appearance with the soy moisturizer compared to those parameters with the control (*p* ≤ 0.05). Furthermore, all parameters were found to significantly improve relative to the baseline with soy moisturizer treatment after 3 months of use (*p* ≤ 0.05) [21].

Similarly, a 2014 presentation at the International Pigment Cell Conference assessed the efficacy of a soy extract and a niacinamide cosmetic serum on the improvement of skin hyperpigmentation, noting the influence of PAR-2 [22]. Briefly, 33 female Korean subjects with skin tone unevenness and facial hyperpigmentation applied the serum twice daily for 2 months, 29 of which completed the trial. Subjects demonstrated significant improvements in all clinical grading attributes at 2 months: overall fairness, skin tone evenness, spot color, blotchiness, smoothness, moisture, and radiance. Furthermore, improvements were observed in skin moisture measurements, in addition to overall quality of life through subjective assessment. After 2 months of serum application, a significantly lower expression of PAR2 was demonstrated with immunohistochemistry analysis. These results suggest that soy extract and niacinamide in a cosmetic serum may be efficacious in the improvement of skin hyperpigmentation, fairness, and radiance parameters, with one of the mechanisms potentially mediated by PAR2 inhibition. However, it is important to note that the efficacy of the serum may be due to inclusion of niacinamide, which also has skin-lightening properties [23]. Furthermore, the authors omitted a vehicle control group and future studies should consider the use of a vehicle control group.

#### 3.3.2. Dermal Network Composition

It is possible that the clinical efficacy of topical soy application may also be mediated by changes in dermal network composition. A 2010 study utilized a multiphoton tomograph DermaInspect device to measure changes in dermal network structure among 24 female subjects following a 3-month application of a cosmetic emulsion containing soy and jasmine [24]. This emulsion was randomized among the arms so that the cosmetic emulsion was applied twice daily to one arm while the other arm was treated with a vehicle. The authors measured autofluorescence signals of the extracellular matrix (ECM) to determine compositional changes over time. While no change was observed with the application of the control cream, treatment with active soy and jasmine ingredients resulted in an enhanced signal of the ECM at week 12, with a greater increase in deeper regions than in superficial ones. The change in extracellular signal indicated modification of dermal composition, such as collagen and/or elastin structure, proportion, or density. However, the study design did not allow for the analysis of specific dermal composition changes, but rather solely demonstrated that compositional changes had occurred.

However, prior studies have observed increased collagen content following soy-based topical application [25]. A study conducted in 1999 assessed the effects of a 2% soya biopeptide encapsulated in lecithin liposomes on collagen content among 10 women in a split-face study [25]. A control emulsion without active soy biopeptides was applied to the left side of the face, while the treatment emulsion was applied to the right side of the face twice daily for 4 weeks. Active treatment stimulated an increase in extractable collagen content among seven patients. However, the authors noted great inter-individual variation in collagen content following extraction.

Another study assessed the effect of 2% soy extract application twice daily for 2 weeks on the dermal papilla number per area in a self-controlled study [26]. Briefly, 21 subjects applied the test cream to one volar forearm and a placebo cream to the other. Flattening of the dermal–epidermal junction, a biological feature associated with aging skin, is mediated by a reduction in the dermal papilla number. However, the authors observed a significant 21% increase in the dermal papilla index following soy extract application, measured via a confocal laser scanning microscope. Thus, authors concluded that soy extract topical application may rejuvenate the structure of aging skin.

#### 3.3.3. Skin Hydration

Six studies assessed topical soy-based application on skin hydration or dryness, two of which used the same topical product: a novel lipid-rich moisturizing body wash (LRMBW) with fatty acids found in skin and soybean oil [27,28]. In 2011, 27 skin of color (SOC) subjects with moderate visual hyperpigmentation applied the LRMBW for three weeks and were assessed for changes in dryness, ashiness, and TEWL [27]. Following three-week daily application, authors observed significant improvement in dryness on outer forearms, elbows, and legs; a significant reduction in skin hyperpigmentation on the outer forearms; and a significant improvement in TEWL on all forearm and outer leg sites. Furthermore, subjects perceived significant improvement in moisturization, softness, and smoothness. A distinct study assessed a 1-month daily application of the LRMBW among 30 women with moderate visual dryness and self-perceived itching [28]. Compared to baseline, authors reported significant improvement in visual dryness and self-perceived itching. However, both studies were open-label and uncontrolled, and may have been susceptible to subject or evaluator bias.

A 2000 double-blind, self-controlled study assessed the effect of a cream containing boswellic acids, silybin and *Centella asiatica* extracts with lyso-phospholipids and soybean non-saponifiable lipids on skin hydration, irritation, and desquamation [29]. Briefly, 20 women enrolled in the study and applied the test cream on one arm and a placebo cream on the other arm twice daily for one month. Skin hydration was measured with Corneometer CM 820 PC. Treatment resulted in a significant improvement in skin extensibility, firmness, and hydration (*p* < 0.02), and no adverse reactions were reported. Lastly, a 2016 self-controlled trial observed a significant increase in stratum corneum hydration with soy oligopeptide application following UVB-induced erythema (*p* < 0.05) [30]. Results were significant on day 1, but not on days 3 and 10. This study is discussed in more detail in Section 3.3.5.

In 2014, authors compared the efficacy of peel-off face masks prepared with polyvinyl alcohol with and without fermented soybean extract versus oil-in-water emulsions with and without fermented soybean extract on skin hydration. In total, 10 healthy Caucasian females between the ages of 18 and 60 years were included in the study. Briefly, 6 separate 5.0 × 5.0 cm sites were chosen on each subject’s volar forearm (three sites on each forearm), where they received one of the four preparations. For face mask sites, a 500 mg test solution was weighed and spread over each site with a 1 mm film thickness; the dry film was washed after 20 min. The oil-in-water emulsion was weighed and delivered using a syringe. Baseline measurements included TEWL, skin hydration, and viscoelastic properties, which were measured again at 45 min, 90 min, and 180 min. The formulations of face masks resulted in a significant increase in hydration compared to that with oil-in-water emulsions. However, the inclusion or absence of soybean extract did not affect results [20]. As the use of fermented soybean extract had no significant effect on skin hydration, or TEWL, the authors concluded there was no benefit of fermented soybean extract in the formulations, though a larger trial is warranted. Lastly, the 2014 open-label trial conducted by Park et al. observed continuous increases in surface and inner moisture throughout the 2-month study [22]. The product assessed contained soy extract and niacinamide and was applied twice daily.

#### 3.3.4. Skin Barrier Status

Six studies reported outcomes implicated in skin barrier status following topical soy-based treatment, including TEWL and pH. As previously described, Feng and Hawkins observed a significant improvement in TEWL on all forearm and outer leg sites following LRMBW application among 27 SOC subjects [27]. Similarly, a 2012 self-controlled study assessed the effects of topical soybean oil on TEWL 30 min after application among six subjects [31]. Petrolatum and various other oils were similarly assessed on different sites. Soybean oil resulted in a significant decrease in TEWL 30 min after application relative to that at the baseline (*p* < 0.05).

While this study depicted the effect of soybean oil on the skin barrier within the same day of application, a separate study assessed the effect of a moisturizer containing soybean extracts on TEWL and skin pH after one month of application [32]. The study specifically enrolled patients with end-stage renal disease (ESRD), as pruritus is common among chronic kidney disease patients. Following a one-month topical application, authors noted an improvement in both TEWL and skin pH measurements, although the poster abstract did not specify whether or not improvements were significant. Lastly, the study that required the application of soy oligopeptide following UVB-induced erythema observed no difference in TEWL measurements between groups at any time points [30]. This study will be discussed in further detail in the Section 3.3.5.

Similarly, the study that assessed face masks and oil-in-water emulsions found no significant difference in TEWL measurements between preparations that included soybean extract and those that omitted the extract [20]. In addition, there was no significant difference between face masks and emulsions. While intra-group analysis revealed significant changes in TEWL measurements over time, such changes were consistent across all four formulations [20]. Authors concluded there was no benefit of fermented soybean extract for TEWL measurements.

Another study assessed the effect of a serum containing multiple ingredients such as vitamin E, niacinamide, and *Glycine max* seed polysaccharides on skin TEWL, in addition to roughness and lipoperoxidation [33]. The researchers recruited 14 female Caucasian subjects aged between 24 and 64 years and instructed them to apply the serum twice daily for 30 days to one arm, with the contralateral arm serving as a control. The authors obtained a baseline TEWL measurement from post-treatment skin and control skin. Next, the skin of both arms was exposed to a 2% sodium laureth sulphate (SLS) solution for 2 h. After SLS exposure, a TEWL measurement was obtained from both arms after 2.5 h and 24 h. However, no significant differences in TEWL measurements were observed on either arm compared to those at the baseline; however, the treated skin demonstrated a smaller change in TEWL following SLS exposure [33]. The authors also assessed skin roughness and lipoperoxidation of the stratum corneum, an indication of antioxidant status. While no significant skin roughness results were observed, authors noted significant lipoperoxidation reduction in the stratum corneum following UV radiation exposure with the treatment compared to that with the control [33].

#### 3.3.5. Erythema Index

Two studies observed protective effects of soy-based topical formulas against UVB-induced erythema. A 2005 study exposed six healthy volunteers to UVB radiation to induce erythema on six circular sites of the ventral forearm [34]. Two control patches were left untreated, and the remaining patches underwent topical treatment with soybean-germ oil or tocopherol acetate. After 3 h, the erythema index of each patch was measured. While tocopherol acetate resulted in a 21.5% inhibition of erythema, soybean germ oil resulted in a 46.8% inhibition of erythema, over twice that of tocopherol acetate [34]. The authors concluded that soybean germ oil exhibits significant UVB-induced erythema protection.

Similarly, a 2016 study subjected nine volunteers to UVB irradiation and assessed the effects of subsequent 2.5 IU/mL, 5.0 IU/mL, and 10.0 IU/mL soy oligopeptide topical treatment compared to those with a vehicle control [30]. One patch remained untreated, solely exposed to UVB. Authors observed a significant decrease in the erythema index with 5 IU/mL or 10 IU/mL topical treatment compared to that with untreated areas (*p* < 0.05). A significant increase in stratum corneum hydration was observed on day 1, but not days 3 and 10, with 5 IU/mL or 10 IU/mL oligopeptide treatment compared to that of untreated areas (*p* < 0.05). There were no significant differences observed with 2.5 IU/mL treatment, suggesting a dose-dependent protective effect of topical soy oligopeptides [30]. It is necessary to note that this publication was ultimately retracted due to the duplicated images contained within the article. However, we included the results in this review, as we felt the duplicated images did not impact evidence quality.

#### 3.3.6. Eczema

A 2010 study evaluated the LRMBW with fatty acids found in the skin and soybean oil among subjects with mild to moderate eczema [35]. Subjects replaced their normal cleanser with the study cleanser for four weeks. The outcome measures included eczema area and severity index (EASI), evaluated by a dermatologist, and self-perceived efficacy. The authors stated that the LRMBW was compatible with other eczema treatments and was perceived to be mild, moisturizing, and appropriate for sensitive skin. However, the poster abstract did not further detail results pertaining to EASI scores.

#### 3.3.7. Acne

In 1996, seven open-label, single-center trials were conducted by Ghyczy et al., during which the effects of topical phosphatidylcholine were assessed among 77 subjects with acne vulgaris [36]. The topical formulation consisted of a liposome containing a 10% lecithin fraction with 80% phosphatidylcholine from soybeans. Subjects applied the formulation to one half of the face daily for 20 days to 2 months, depending on the trial. The other side of the face was utilized as a control, to which no product was applied. By day 28, the average comedone counts, combined from all seven trials, decreased by 64.3% with treatment, while no reduction was noted on the control side. Additionally, the efflorescence counts decreased by 75% with treatment compared to the decrease of 0% on the control side [36]. These results suggest that soybean phosphatidylcholine may be beneficial for the treatment of acne. However, individual findings from each of the seven studies are not listed and the statistical significance of results are not described.

#### 3.3.8. Hair

One open-label, uncontrolled study evaluated extracts from 13 herbs including black soybean and green tea on hair parameters among 10 adults [37]. While the subjects were concerned about hair loss, none had been officially diagnosed with alopecia. Subjects applied the extract topically for 10 min daily for three months. The authors observed a significant improvement in mean lost hair compared to that at the baseline (140.7 vs. 38.8, *p* = 0.002). Similarly, the authors observed a significant improvement in the mean diameter of lost hair (72.4 µm vs. 80.4 µm, *p* = 0.022) [37]. Thus, they concluded that the extract may promote hair growth and prevent hair loss, although a double-blind, placebo-controlled trial is necessary to further assess efficacy.

**Table 2:** Summary of clinical studies assessing soy-based topical application on dermatologic outcomes.

Intervention (*n*)	Dose and Duration	Control (*n*)	Subjects	Outcome	Major Results	Jadad Score	Author (Year), Study Design
Soy moisturizer containing STI and BBI (31)	Twice daily for 3 mo	Vehicle control (32)	Women with moderate photodamage	Skin aging parameters	-Significant improvement in mottled pigmentation, blotchiness, dullness, fine lines, overall texture, skin tone, and appearance with soy moisturizer compared to those parameters with to the control (*p* ≤ 0.05) -All parameters depicted a significant improvement from baseline with soy moisturizer treatment (*p* ≤ 0.05)	4	Wallo et al., (2007), RCT [21]
Serum containing soy extract and niacinamide (29)	Twice daily for 2 mo	N/A	Women with skin tone unevenness and facial hyperpigmentation	Skin parameters including tone evenness	-Significant improvement in overall fairness, skin tone evenness, spot color, blotchiness, smoothness, moisture, and radiance -Increase in surface and inner moisture throughout the study		Park et al., (2014), open-label, uncontrolled [22]
Emulsion containing soy and jasmine (24)	Twice daily for 3 mo	Vehicle cream (24, self-controlled)	Women	Dermal network composition (measured via multiphoton tomograph DermaInspect device)	-Autofluorescence signal of ECM enhanced after 3 mo treatment (indicating collagen and/or elastin dermal modification) -Greater signal increase in deeper regions vs. superficial -No change in signal after 3 mo control	N/A	Bazin et al., (2010), self-controlled [24]
2% soya biopeptide encapsulated in lecithin liposomes (10)	Twice daily for 1 mo	Placebo emulsion (10, self-controlled)	Women	Collagen content	-7/10 patients depicted increased collagen content -Great inter-individual collagen content variation	N/A	Andre-Frei et al., (1999), self-controlled [25]
Emulsion containing 2% soy extract (21)	Twice daily for 0.5 mo	Placebo cream (21, self-controlled)	Women	Dermal papilla index	-Significant 21% increase in the papilla index with soy extract 1 compared to that with placebo-treated control	N/A	Südel et al., (2005), self-controlled [26]
LRMBW with fatty acids found in skin and soybean oil (27)	Daily for 0.75 mo	N/A	SOC women with moderate visual skin ashiness	Dryness, ashiness, TEWL, and self-perceived assessment	-Significant improvement in dryness on outer forearms, elbows, and legs after 0.75 mo -Significant reduction in skin ashing severity on the outer forearms -Significant TEWL improvement on all forearm and outer leg sites -Subjects perceived significant improvement in moisturization, softness, and smoothness -Subjects perceived significant reduction in ashy appearance, itch, and skin tightness	N/A	Feng and Hawkins (2011), open-label, uncontrolled [27]
LRMBW with fatty acids found in skin and soybean oil (30)	Daily for 1 mo	N/A	Women with moderate visual dryness and self-perceived itch	Visual dryness and self-perceived itching	-Significant improvement in visual dryness and self-perceived itching from baseline	N/A	Foy et al., (2010), open-label, uncontrolled [28]
Cream containing boswellic acids and Silybin and *Centella asiatica* extracts with lyso-phospholipids and soybean non-saponifiable lipids (20)	Twice daily for 1 mo	Placebo cream (20, self-controlled)	Women	Dryness, irritation, and desquamation	-Significant improvement in skin extensibility and firmness with treatment (*p* < 0.02) -Significant increase in skin elasticity and hydration with treatment (*p* < 0.02) -No adverse reactions reported	N/A	Martelli et al., (2000), self-controlled [29]
Soy oligopeptides following UVB-induced erythema (9) *	2.5 IU/mL, 5 IU/mL, or 10 IU/mL applied for five minutes following irradiation (3 consecutive days)	Negative control, vehicle control (9, self-controlled)	Men	EI, SC hydration, and TEWL	-Significant decrease in EI with 5 IU/mL or 10 IU/mL topical application compared to that of sites solely undergoing irradiation (*p* < 0.05) -Significant increase in SC hydration with 5 IU/mL or 10 IU/mL topical application compared to that of sites solely undergoing irradiation (*p* < 0.05) on day 1, but not days 3 and 10 -No significant difference in TEWL between groups at any time points	N/A	Zhou et al., (2016), self-controlled [30]
-Peel-off face mask in PVA with soybean extract -Oil-in-waste emulsion with soybean (10)	-Facial masks and emulsions consisting of 500 mg spread over a 5 × 5 cm forearm site for 3 h	-Peel-off face mask in PVA without soybean extract -Oil-in-waste emulsion without soybean (10, self-controlled)	Women	Skin firmness, hydration, and TEWL	-Significant increase in hydration of upper skin layers with face mask compared to emulsion; hydration was not affected by presence of soybean extract -No significant difference in skin firmness between face mask vs. emulsion, or between that with soybean extract vs. without -No significant difference in TEWL between face mask vs. emulsion, or between that with soybean extract vs. without	N/A	Velasco et al., (2014), self-controlled [20]
Soybean oil (6)	2 mg/cm^2^ for 30 min	Petrolatum; various other oils were also assessed (6, self-controlled)	Men and Women	TEWL	-Significant decrease in TEWL 30 min following soybean oil application (*p* < 0.05)	N/A	Patzelt et al., (2011), self-controlled [31]
Moisturizer containing soybean extracts (*n* unknown)	Topical application for 1 mo	N/A	Patients with ESRD	TEWL, skin pH, PAR2 expression, and pruritus severity	-Improvement in barrier status (TEWL and skin pH) with moisturizer	N/A	Kim et al., (2010), open-label, uncontrolled [32]
Proteum serum containing *Glycine max* seed polysaccharides (14)	Twice daily for 1 mo	No topical applied to contralateral arm (14, self-controlled)	Women	Skin roughness, TEWL, and stratum corneum lipoperoxidation	-Non-significant decrease in skin surface roughness with treatment at 1 mo -No significant difference in TEWL following SLS exposure compared to baseline for both treatment and control -Significant reduction in lipoperoxidation in the stratum corneum following UV irradiation with treatment compared to control	N/A	Barba et al., (2017), self-controlled [33]
Soybean germ oil following UVB-induced erythema (6)	200 μL topically applied for 3 h	Negative control and tocopherol acetate (6, self-controlled)	Men and Women	EI	-46.8% erythema inhibition with soybean germ oil -21.5% erythema inhibition with tocopherol acetate	N/A	Bonina et al., (2005), self -controlled [34]
LRMBW with fatty acids found in skin and soybean oil (n unknown)	Daily for 1 mo	N/A	Subjects with mild to moderate eczema	EASI and self-perceived assessment	-Treatment was compatible with subjects’ prior eczema treatments -Subjects perceived LRMBW to be mild, moisturizing, and appropriate for sensitive skin	N/A	Zhang et al., (2010), open-label, uncontrolled [35]
Lecithin fraction containing 80% phosphatidylcholine (77)	1 mg/cm^2^ applied once daily to half of face for 20 days to 2 mo	No topical supplement applied to contralateral face (77, self-controlled)	Children 13–18 with acne vulgaris	Comedone and efflorescence counts	Meta-analysis of 7 single-center trials: -40% and 50% reduction in comedone and efflorescence count, respectively, with treatment compared to 0% and 6.7% reduction on control side (day 14) -64% and 75% reduction in comedone and efflorescence count, respectively, with treatment compared to 0% and 0% reduction on control side (day 28)	N/A	Ghyczy et al., (1996), self-controlled [36]
Extract from 13 herbs including black soybean and green tea (10)	10 min application daily for 3 mo	N/A	Men and women (no official alopecia diagnosis)	Hair parameters	-Mean number of lost hairs: 140.7 (SD 59.4) at baseline vs. 38.8 (SD 54.4) at 3 mo (significant improvement, *p* = 0.002) -Mean diameter of lost hair: 72.4 µm (SD 11.9) at baseline vs. 80.4 µm (SD 8.8) at 3 mo (significant improvement, *p* = 0.022)	N/A	Sung and Kim (2022), open-label, uncontrolled [37]

Abbreviations: BBI = Bowman–Birk protease inhibitor; ECM = extracellular matrix; EI = erythema index; ESRD = end-stage renal disease; LRMBW = lipid-rich moisturizing body wash; Mo= month; PVA = polyvinyl alcohol; SC = stratum corneum; SD = standard deviation; SLS = sodium laureth sulphate; SOC = skin of color; STI = soybean trypsin inhibitor; UV = ultraviolet; UVB = ultraviolet B. * Retracted publication due to duplicated images [30].

## 4. Discussion

Soy isoflavones, polyphenols found in soybean, have been shown to exhibit a variety of physiological properties, such as antioxidant, anticancer, antimicrobial, anti-inflammatory, anti-menopausal osteoporosis, and anti-aging properties [38,39]. While precise mechanisms remain unclear, recent evidence has demonstrated the potential efficacy of isoflavone supplementation in preventing chronic diseases characterized by inflammation [39]. Furthermore, their phytoestrogen and antioxidant properties have been hypothesized to underly their efficacy in the prevention and treatment of various diseases [38]. Acne, hair health, and skin aging have emerged as dermatologic targets for soy isoflavone supplementation or application.

Briefly, 30 studies evaluated the use of oral or topical soy-based formulations for a variety of dermatologic outcomes, including cutaneous aging parameters, skin barrier status, hydration, hyperpigmentation, dermal network composition, erythema, and acne lesion counts. In total, 29 studies observed efficacious results for at least one measured parameter. One study observed no significant difference among formulations including soybean extract vs. those without; the outcomes included skin firmness, hydration, and TEWL, although only ten subjects were enrolled in the self-controlled study [20]. Overall, soy-based supplementation or topical application appears to be efficacious for a variety of purposes. However, many studies evaluated formulations containing other ingredients that are implicated in skin, nail, and hair health. The heterogeneity of formulations used among included studies hinders the ability to draw conclusions on the efficacy of soy alone.

Of the assessed outcomes, parameters implicated in cutaneous aging were most frequently studied. Studies assessing oral supplementation observed reduced wrinkling, both in area and depth, sagging, hyperpigmentation or discoloration, and improvements in elasticity, dryness, and coarseness. One study observed a significant increase in collagen fiber number and elastic fiber number in 25/29 subjects and 22/29 subjects, respectively [10], suggesting that dermal compositional changes may mediate observed improvements in cutaneous aging. Similarly, topical soy-based application significantly ameliorated numerous parameters associated with cutaneous photoaging, such as mottled pigmentation, blotchiness, dullness, fine lines, overall texture, skin tone, and appearance [21]. In addition, three studies collectively suggest that soy-based topical application can modify ECM composition [24], increase collagen content [25], and increase dermal papilla number [26], which may mediate observed amelioration of cutaneous chronological aging and photoaging [21]. Although there were fewer studies evaluating the effects of topical soy application on clinical aging parameters compared to oral soy supplementation, evidence suggests that both routes of soy administration can impact parameters of cutaneous aging.

Skin hydration and barrier integrity were frequently reported outcomes among both oral and topical studies. While Lee and colleagues observed a significant increase in skin hydration on the face and forearm among oral supplementation subjects vs. that with the control [16], two other studies failed to observe significant changes in hydration with oral supplementation [11,12]. However, each study utilized different oral supplementation recipes. Furthermore, while Lee et al. measured skin hydration via Corneometer CM825 [16], Jenkins et al. used TEWL measurements [12], and Ueno et al. used Skicon-200EX [11].

In contrast, all studies assessing topical soy-based application on skin hydration observed significant improvements [20,29,30]. In addition, while both studies [11,12] assessing TEWL following oral soy-based supplementation did not observe significant changes, three out of six studies found a significant TEWL improvement following topical soy application. The three studies failed to observe significant differences measured TEWL following UVB-induced erythema [30], SLS exposure [33], or after brief (3 h) application [20]. It is possible that improved TEWL measurements require more frequent application, although one study observed significant decreases in TEWL measurements following 30 min of soybean oil application [31]. While effects may be dependent on specific formulations and protocols, it appears that improvements in TEWL and thus skin barrier integrity are more likely with topical soy application rather than oral supplementation.

The effect of soy supplementation or topical application on acne vulgaris lesions was assessed in Three included studies [17,18,36], one of which compiled results from seven sub-studies [36]. Both studies assessing oral supplementation observed a significant decrease in mean total lesion count. Furthermore, comedone and efflorescence counts decreased with topical application of phosphatidylcholines from soybeans. Both oral supplementation and topical application appear to have efficacious effects on acne vulgaris lesions, with the effects being likely mediated by both antiandrogenic and anti-inflammatory processes [18]. Prior in vitro studies assessed isoflavone supplementation in prostate tissue to inhibit 5α-reductase and 17β hydroxysteroid dehydrogenase, both of which function in androgen synthesis [40,41]. Riyanto et al. similarly demonstrated antiandrogenic effects in vivo, as authors observed decreased DHT levels following isoflavone supplementation in an amount of 160 mg daily for 3 months [18]. In addition, anti-inflammatory properties may mediate the reduction in papules, pustules, nodules, and total acne vulgaris lesion counts [17].

Other assessed outcomes include hair and nail parameters and protection against UVB irradiation. One study observed decreased hair loss with the topical application of a herbal extract formulation, including black soybean extract [37]. Another study observed significantly decreased roughness and dullness with oral soy supplementation [3]. As soy is a rich source of phytoestrogens, authors suggest that skin, hair, and nail effects of soy supplementation may be mediated by phytoestrogens, especially among postmenopausal women [3]. The efficacy of oral supplementation with 160 mg of soy isoflavones for acne coupled with its ability to inhibit 5α-reductase make a future study in hair growth and hair health warranted.

Lastly, two studies observed a protective effect of topical soy oligopeptides or soybean germ oil following UVB-induced irradiation [30,34]. The authors suggest that soy oligopeptides elicit a protective effect against UVB irradiation due to its large unsaponifiable fraction, providing resistance to thermal oxidation [34]. Furthermore, the positive effects may be mediated through UV absorption and the clearance of oxygen free radicals [30].

Overall, this review highlights the multitude of potential dermatologic applications of soy-based oral and topical products. Soy supplementation or application appears particularly useful for achieving a reduction in wrinkles and other parameters associated with chronological or photoaging, likely mediated by dermal compositional changes. Efficacious results were also observed for specific dermatologic conditions, including acne vulgaris, vulvar lichen sclerosis, and eczema. While many studies were of high evidence quality, such as the RCTs and self-controlled trials, studies with greater sample sizes are warranted. In addition, future studies are required to determine optimal routes of application and optimal formulations for intended outcomes.

## Figures and Tables

**Table 1:** Summary of clinical studies assessing oral soy-based supplementation on dermatologic outcomes.

Intervention (*n*)	Dose and Duration	Control (*n*)	Subjects	Outcome	Major Results	Jadad Score	Author (Year), Study Design
Supplement with soy extract, fish protein, polysaccharides, vitamins C and E, zinc, and extracts from white tea, grape seed, tomato, and chamomile (38)	Four tablets daily for 6 mo (350 mg soy extract daily)	Placebo tablet (42)	Post-menopausal women	Skin aging parameters	-Clinical assessment: significantly greater improvement in forehead, periocular, and perioral wrinkles; facial skin laxity, sagging, and mottled hyperpigmentation; under-eye dark circles, and decolletage and hand crepiness with treatment compared to control at mo 6 (*p* < 0.05) -Photo assessment: significantly greater improvement in forehead and periocular winkles, facial mottled hyperpigmentation, and overall appearance with treatment compared to control at mo 6 (*p* < 0.05)	4	Skovgaard et al., (2006), RCT [8]
Test food containing soy isoflavone aglycone (13)	40 mg soy isoflavone aglycone daily for 3 mo	Placebo food (13)	Women	Skin aging parameters	-Significant improvement in fine wrinkles (*p* < 0.05) and of malar skin elasticity (*p* < 0.05) with test food compared to placebo at mo 2 -Significant intragroup improvement of skin microrelief at the lateral angle of eyes with treatment (*p* < 0.05), although there was no significant difference compared to control	4	Izumi et al., (2007), RCT [9]
Soy supplement (20)	20 g soy protein/160 mg isoflavones once daily for 6 mo	Self-selected diet (20)	Women with mild to moderate photoaging	Skin, hair, and nail parameters	-Significant investigator-assessed improvement in facial skin flaking (*p* = 0.028), discoloration (*p* = 0.045), and overall appearance (*p* = 0.05), and hair roughness (*p* = 0.041), manageability (*p* = 0.018), and overall appearance (*p* = 0.016) in the soy supplement group at 3 mo -Significant investigator-assessed improvement in facial skin wrinkling (*p* = 0.004), discoloration (*p* = 0.016), and overall appearance (*p* = 0.0001); hair roughness (*p* = 0.004), dullness (*p* = 0.048), and overall appearance (*p* = 0.005); and nail roughness (*p* = 0.017), ridging (*p* = 0.006), flaking (*p* = 0.049), splitting (*p* = 0.007), and overall appearance (*p* = 0.008) at 6 mo	2	Draelos et al., (2007), RCT [3]
Supplement containing isoflavones and concentrated soy (29)	100 mg daily for 6 mo	N/A	Post-menopausal women	Skin aging parameters	-23 subjects depicted a 9.46% increase in epidermal thickness (*p* < 0.01) -Mean increase in wrinkling of 10.4% ± 1.9% -25 subjects depicted a significant increase in collagen fiber number (7.6% ± 1.5%, *p* < 0.01) -22 subjects depicted a significant increase in elastic fiber number (18.8 ± 4.8%, *p* < 0.01)	N/A	Accorsi-Neto et al., (2009), open-label, uncontrolled [10]
5-equol supplement developed by whole soy germ fermentation with a strain of equol-producing lactic acid bacteria (34 subjects supplemented with 10 mg and 33 subjects supplemented with 30 mg)	10 mg or 30 mg daily for 3 mo	Placebo supplement (34)	Post-menopausal women	Skin aging parameters	-Significant wrinkle area reduction in both treatment groups compared to control (*p* < 0.05) -Significant difference in wrinkle depth with 30 mg treatment compared to control (*p* < 0.05) -No significant differences between groups for TEWL, hydration, and elasticity	5	Ueno et al., (2011), RCT [11]
Cocktail with soy isoflavones, lycopene, vitamins C and E and an ω-3 fish oil capsule (51 subjects supplemented with treatment 1, which consists of 70 mg isoflavones, and 53 subjects supplemented with treatment 2, which consists of 40 mg isoflavones)	One dose daily for 3.5 mo	Placebo cocktail and capsule (55)	Post-menopausal women	Skin aging parameters	-Rz value significantly increased from baseline in the control group and did not significantly change in either treatment groups -Significantly greater change in Rz value in the control group vs. that in treatment 1 (*p* = 0.0045) and treatment 2 (*p* = 0.0081) -No significant difference in skin firmness, elasticity, and TEWL between placebo and treatment groups at any time points -17.3% of treatment 2 subjects depicted an increase in collagen vs. 3.6% control subjects (*p* = 0.0259)	5	Jenkins et al., (2013), RCT [12]
Fermented soymilk containing isoflavone aglycones and probiotic LcS (27)	100 mL fermented soymilk, 32.5% isoflavone aglycones twice daily for 2 mo	100 mL fermented soymilk without isoflavone aglycones (25)	Premenopausal women	Skin condition and gut microbiome	-Both groups depicted improved skin conditions parameters from scores of satisfaction, dryness, moisture, elasticity, coarseness, pigmentation, and/or stratum corneum morphology; no significant difference between groups -Significant decrease in *Enterobacteriaceae* and *Porphyromonadaceae* abundance with treatment during intake period -No significant difference in urinary isoflavone levels between both groups at any time point, although both groups demonstrated increased urinary isoflavones during intake period	5	Nagino et al., (2018), RCT [13]
Nutraceutical with *Glycine max*, *Cimicifuga racemose*, *Vitex agnus-castus*, and *Oenothera biennis* extract (50)	One tablet daily for 3 mo	Soybean oil-containing placebo tablet (51)	Post-menopausal women	Skin aging parameters	Significant improvement in skin elasticity (Cohen’s *d* = 1.56), roughness (*d* = 1.53), smoothness (*d* = 1.33), scaliness (*d* = −0.8), and wrinkles (*d* = −1.02) with treatment compared to control at 3 mo	5	Tumsutti et al., (2022), RCT [14]
Supplement containing 98% S-equol (isoflavone derivative), 2% daidzein, 0.2% glycitein, and 0.1% genistein extracted from fermented soybeans (27)	10 mg daily for 3 mo	Placebo control (30)	Postmenopausal women	Skin autofluorescence (an indicator of AGEs, and a contributor in aging)	-No significant difference between groups for skin autofluorescence and visceral fat at 3 mo -Skin autofluorescence improved in 3/18 (16.7%) with no equol exposure; 7/20 (35%) with extrinsic exposure; 3/8 (37.5%) with intrinsic exposure; and 3/4 (75%) with intrinsic and extrinsic exposure -Significant improvement in climacteric symptoms with treatment compared to control (*p* = 0.045)	4	Yoshikata et al., (2021), RCT [15]
Cocktail containing barley and soybean formula (32)	3 g in 100 mL daily cocktail for 2 mo	Placebo cocktail (33)	Healthy volunteers with dry skin	Skin hydration	-Significant increase in skin hydration on the face (mo 1 and 2) and forearm (mo 1) with treatment compared to control	1	Lee et al., (2015), RCT [16]
Isoflavone supplement (5 subjects supplemented with 80 mg tablets, 5 subjects supplemented with 120 mg tablets, and 5 subjects supplemented with 160 mg tablets)	40 mg, 80 mg, 120 mg, or 160 mg daily for 1 mo	Placebo supplement (5)	Women with acne vulgaris	Acne vulgaris lesion number	-Significant decrease in mean total acne vulgaris lesions from baseline in the treatment group receiving 160 mg (*p* < 0.05) -No significant decrease in mean total acne vulgaris lesions from baseline in other treatment groups	2	Riyanto and Subchan (2015), RCT [17]
Soybean isoflavone supplement (20)	160 mg daily for 3 mo	0 mg placebo (20)	Women with mild to severe acne vulgaris	Acne vulgaris lesion number and DHT	-Acne lesion number significantly decreased from baseline with treatment (*p* < 0.05) -Significant difference in acne lesion number change between both groups (*p* < 0.05) -While DHT levels increased mean 97.4 pg/mL in the control group, DHT levels decreased 145.6 pg/mL in the treatment group	3	Riyanto et al., (2015), RCT [18]
Oral and topical ASE containing vitamin E, PABA, and phytosterols (23)	300 mg daily for 3 mo and topical ASE application twice daily for 6 mo	N/A	Women with mild to moderate vulvar lichen sclerosis	GSS75 and GOS75	-88.9% and 72.2% reached GOS50 and GOS75, respectively at 6 mo -100% and 70.6% reached GSS50 and GSS75, respectively at 6 mo -Itching and burning significantly decreased (*p* < 0.00001) -Dyspareunia significantly decreased (*p* = 0.003)	N/A	Borghi et al., (2015), open-label, uncontrolled [19]

Abbreviations: AGEs = advanced glycation end products; ASE = avocado and soybean extracts; DHT = dihydrotestosterone; GOS50 = global objective score of ≥50%; GOS75 = global objective score of ≥75%; GSS50 = global subjective score of ≥50%; GSS75= global subjective score of ≥75%; LcS = *Lactobacillus casei Sherota*; Mo = month; PABA = para-aminobenzoic acid; Rz = peak to valley distance; TEWL = Transepidermal water loss.

## Data Availability

The data used to support these findings are included in the article. No additional data is available.

## Abbreviations

ASE	Avocado and soybean extracts
BBI	Bowman–Birk protease inhibitor
DHT	Dihydrotestosterone
EASI	Eczema area and severity index
ECM	Extra-cellular matrix
FSM	Fermented soy milk
GOS	Global objective scores
GSS	Global subjective scores
LcS	*Lactobacillus casei* Sherota
LRMBW	Lipid-rich moisturizing body wash
PAR-2	Protease-activated receptor 2
RCT	Randomized controlled trial
SLS	Sodium laureth sulphate
SOC	Skin of color
STI	Soybean trypsin inhibitor
TEWL	Transepidermal water loss
UVB	Ultraviolet B
